# Occurrence of mollicutes infection and molecular identification of ureaplasmas from the reproductive tract of dairy cattle kept under tropical conditions

**DOI:** 10.1007/s42770-025-01837-0

**Published:** 2026-05-11

**Authors:** Ana Carolina Nunes de Morais, Danielle Regis Pires, Leandro dos Santos Machado, Lucas de Figueiredo Cardoso Barbosa, Pedro Panzenhagen, Mario Felipe Alvarez Balaro, Guilherme Nunes de Souza, Maria Lucia Barreto, Elmiro Rosendo do Nascimento, Nathalie Da Cunha

**Affiliations:** 1 Fluminense Federal University (UFF), Niterói, RJ Brazil; 2 Federal Rural University of Rio de Janeiro (UFRRJ), Seropédica, RJ Brazil; 3 Federal University of Rio de Janeiro (UFRJ), Rio de Janeiro, RJ Brazil; 4 Brazilian Agricultural Research Corporation (EMBRAPA), Juiz de Fora, MG Brazil

**Keywords:** Mollicutes, Bovine reproductive disease, Risk factors, Intergenic 16-23S rRNA

## Abstract

Bacterial diseases associated with the Mollicutes class can lead to decreased milk production and reproductive disorders in cattle, causing significant economic losses and compromising animal welfare. This study aimed to investigate the presence of Mollicutes and *Ureaplasma diversum* in the reproductive tract of dairy cows and determine the associated risk factors, as well as identify circulating strains of *Ureaplasma* in herds. Vaginal swab samples from 392 lactating cows were subjected to generic polymerase chain reaction (PCR) for Mollicutes detection and specific PCR for *U. diversum* identification. An epidemiological questionnaire was used to analyze the risk factors. Positive samples for *U. diversum* were sequenced from the intergenic region 16–23 S rRNA and multiple alignments were performed to generate a phylogram. The geographical coordinates of the municipalities where the herds were located were used for the phylogeographic analysis. Mollicutes and *U. diversum* were detected in 45.4% (178/392) and 21.7% (85/392) of the samples, respectively, with a herd-level prevalence of 88.8%. Age group heifers (OR = 4.006; CI 1.283–12.510) and history of genital lesions (OR = 5.564; CI 3.011–10.284) were the biggest associated risk factors, in addition to other effects. The frequency of these agents was high and different strains of *U. diversum* were found to be spread throughout the studied states, likely due to the extensive animal movement network.

## Highlights

Mollicutes and *Ureaplasma diversum* were detected in 45.4% and 21.7% of genital swab samples.

Prevalence of Mollicutes and *Ureaplasma diversum* per herd were 88.8%.

Risk factors identified for Mollicutes were infection reproductive disorders variables, herds with over 100 animals, heifers and average production over 500 kg.

Different strains of *U. diversum* are spread throughout the studied region, probably due to the intense movement of animals.

## Introduction

Genital diseases associated with infection caused by bacteria in class Mollicutes, mainly due to the genera *Ureaplasma* and *Mycoplasma*, have been reported in cattle herds. Generally, this condition occurs in outbreaks and is associated with economic losses and animal welfare impairment, resulting in infertility, poor reproductive performance and increased cost of treatment [8, 25, 35].

*Ureaplasma diversum* has already been isolated from healthy, with no apparent lesions or previous/subsequent reproductive disease, and sick cattle, with genital disorders. The risk of infections is higher in younger cows, resulting in fewer births. Nevertheless, the infection is not dependent upon the presence of clinical symptoms, since the first isolates were, apparently, in the genital/reproductive tract of healthy cows. The presence of these microorganisms in healthy cattle herds makes it difficult to identify risk factors for infection, which makes establishing infection control strategies more difficult [10, 31]. Genital disorders have been strongly associated with reproductive diseases such as granular vulvovaginitis (GVV), a syndrome characterized by vulvar discharge, hyperemia, granules and vesicles in the genital mucosa. Salpingitis, endometritis, vulvitis, placentitis, fetal alveolitis, infertility, dystocia, the birth of weak calves, and abortion (middle to late pregnancy) also have been reported [23, 15, 16, 35]. *Ureaplasma diversum* is considered sporadic and not commonly investigated within the differential diagnosis of reproductive disorders and abortions. It is still unclear whether the low detection rates are due to the low prevalence or under-investigation by routine laboratory diagnosis. In addition, little is known about physiopathology, assuming that such an agent’s presence in the vulva or vestibule of the vagina is usual and isolation in the cervix or uterus can signify a pathological condition [10].

The emerging genital diseases associated with Mollicutes led to research on the detection of these microorganisms in the reproductive tract of cattle worldwide. In Central America, León et al. [19] isolated *U. diversum* in heifers and dairy cows from Costa Rica. In Asia, Chandra et al. [9] isolated Mollicutes from both sick (with GVV) and healthy cows, with *U. diversum* being more frequent in repeat-estrus cows from India. Similarly, in Europe, Díaz et al. [10] detected *U. diversum* in animals with and without GVV from herds with a history of abortion from Spain. Australia is also not free of this agent once Argue et al. [3] detected *U. diversum* in cattle herds with a reproductive disease history. In South America, Sosa et al. [32] isolated *U. diversum* and *M. bovigenitalium* in healthy bulls from Argentina, which is of great relevance due to the possibility of venereal transmission. In Brazil, the association of Mollicutes with genital diseases is not well investigated. Since the first detection of *U. diversum* in cattle [8], new studies reported this agent in healthy and sick animals in several regions with frequencies ranging from 63.7% to 65.6% for Mollicutes and from 13% to 41.6% for *U. diversum* [4, 6, 15, 21, 24, 26, 29, 30].

Since Mollicutes and *U. diversum* are disease-causing agents influenced by the herds management practices, identifying ureaplasma strains circulating in different herds is relevant for mitigating economic losses in cattle production. In this context, this study investigated the presence of Mollicutes and *U. diversum* in the reproductive tract of dairy cows and determined the risk factors associated with infection.

## Materials and methods

This study was approved by Ethics Committee for the Use of Animals of the Universidade Federal Fluminense (number 987/2017) and was conducted under the principles of the Brazilian Society of Laboratory Animal Science, which regulates conditions for trials involving animals.

### Study design

Sampling to obtain the minimum number of animals was based on the formula n = Z^2^ *x* P(1-P)/E^2^ [33], where Z refers to the confidence interval, P is the estimated prevalence, and E is the error. In the current study, the Z value was 95%, the P value 50% (since the prevalence of genital infection by Mollicutes or *Ureaplasma diversum* in the region is unknown) and the E value 5%, obtaining a minimum sample of 384 animals to be analyzed. Herds were selected by convenience sampling (non-probabilistic sampling), and animals from each herd were randomly selected.

### Origin of animals

**Fig. 1:**
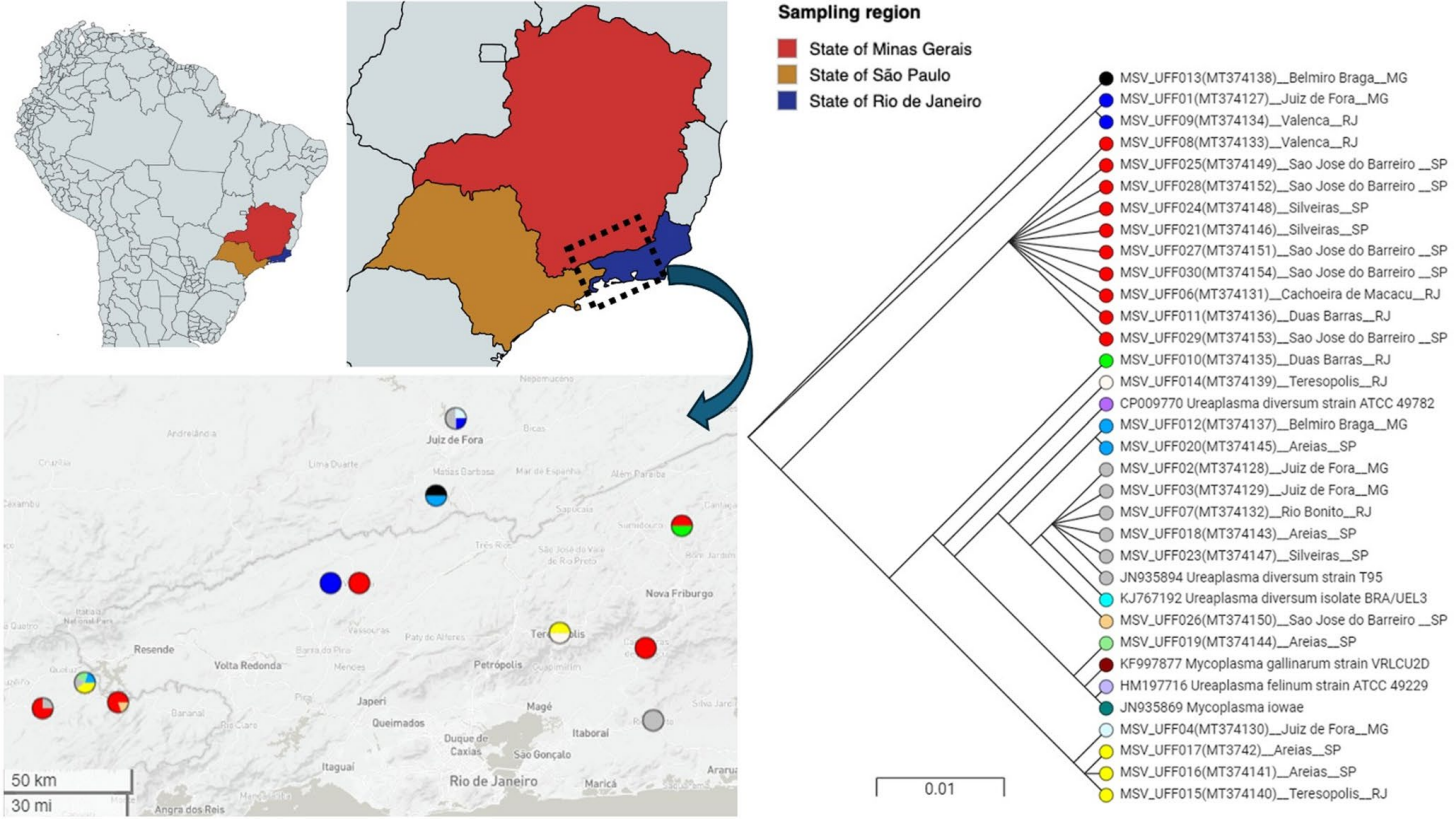
Phylogenetic tree and geographic distribution of Ureaplasma diversum strains from dairy cattle in Southeast Brazil. Colored circles indicate clusters identified in this study. The map shows the origin of isolates, with colors corresponding to the phylogenetic clusters

In this cross-sectional study, samples of 392 animals were obtained from dairy cattle herds raised under tropical conditions in Southeast Brazil between March 2018 and July 2019 (Fig. 1).

### Sampling

Vaginal mucosa swab samples were collected from apparently healthy reproductive females of different ages, breeds, and stages of lactation. Sampling was conducted by rubbing sterile swabs on the internal vaginal wall after cleaning the external vulvar region with 70% ethyl alcohol. Swabs were collected in duplicates: one in tubes containing 3.0mL of modified Frey medium (adapted from Frey et al. [14] and the other in 3.0mL of UB medium for *Ureaplasma* spp [28]. The material was stored in isothermal boxes, transported under refrigeration (4 °C), and subsequently kept at − 20 °C until laboratory analyses.

### DNA extraction and polymerase chain reaction (PCR)

The extraction of genomic DNA was performed from 500µL of each sample using the thermal extraction method adapted from Fan et al. [12], with Tris EDTA pH 8.0 buffer being the DNA eluent. The extracted DNA was evaluated in a Biodrop^®^ UV/VIS spectrophotometer (Biochrom^®^ Ltd., Cambridge, UK), regarding the concentration (ng/µL) and the degree of purity. The extracted DNA from the collection in the modified Frey medium was used to perform the PCR for the detection of the microorganisms in class Mollicutes, using the GPO3/MGSO primers that are capable of amplifying a 270 bp fragment of the 16S rRNA gene and thermal cycling according to Van Kuppeveld et al. [34]. The total reaction volume was 25µL, containing reaction buffer (10mM Tris HCl pH 8.0) 1×, 2.0mM MgCl_2_, 0.2mM dNTP, 0.2 µM primers, and 1.0U of Taq Polymerase (Ludwig^®^). For *U. diversum* detection, PCR was performed with the DNA obtained from materials packed in UB medium for *Ureaplasma* spp., with thermal profile and the internal primers UD3 and UD4 used in the nested PCR as described by Buzinhani et al. [6], which amplifies a 215 bp fragment of the 16S rRNA gene. The total reaction volume was 25µL, containing 1× reaction buffer (10mM Tris HCl pH 8.0), 1.5mM MgCl_2_, 0.2mM dNTP, 0.5µM primers, and 1.0U of Taq Polymerase (Ludwig^®^). Standard strains of *Mycoplasma bovis* Donetta PG 45 and *U. diversum* ATCC 49,782 were positive controls for each reaction, while ultrapure water was used as a negative control. For all the reactions, 100ng of DNA template was used. The amplified products were submitted to an electrophoretic run at 90mV, on a 1.5% agarose gel, added with 0.025µL/mL of ethidium bromide and a 100 bp molecular marker. The gel was visualized in a UV light transilluminator and the image was captured for photo documentation.

### Genomic sequences analysis

Polymerase chain reaction (PCR) was performed by selection of the primer pair (UdivF/UdivR) targeting the 16–23 S rRNA internal transcribed spacer (ITS) region of the *U. diversum* [35]. We used the DNA sequence analyzer ABI3730xlv (Thermo Fisher Scientific, Waltham, Massachusetts, USA) for sequencing the forward and reverse amplicons. The complete ITS sequences from *U. diversum* were trimmed for base quality and assembled in contigs with Geneious Prime^®^ 2020.0.4 (https://www.geneious.com) (Biomatters, Ltd., Auckland, New Zealand). The resulting consensus sequences were used as a query for the nucleotide BLAST analysis against the NCBI database to determine the similarity with the species of *U. diversum*. Closely related partial 16 S and 23 S rRNA gene and complete ITS sequences from other organisms were selected from GenBank [5] through a basic local alignment search tool [1] and were used for multiple alignments. All the regions that could not be unambiguously aligned due to length variation were trimmed before alignment. Multiple alignments were performed using the Muscle software 3.8.425 [11]. A phylogram was generated using Geneious Prime^®^ 2020.0.4 tree builder with the sequences concatenated in one single alignment, which included sequences of *Mycoplasma gallinarum* strain VRLCU2D (KF997877) and *Mycoplasma iowae* (JN935869) as outgroups. The neighbour-joining tree method was performed with the genetic distance model Tamura-Nei and the bootstrapping of 10,000 replications. The geographical coordinates of the municipalities where the herds were located were used to perform the phylogeographic analysis employing MicroReact^®^ (http://microreact.org) [2].

### Epidemiological questionnaire

Concomitantly with the material collection, an epidemiological questionnaire with closely related questions was administered in all the properties to investigate health management practices and data on herd reproductive history.

### Statistical analysis

Risk factor analysis was performed using PCR detection of microorganisms within the class Mollicutes (GPO3/MGSO primers) as the dependent variable. This approach is technically and epidemiologically justified because Mollicutes, including the genera *Mycoplasma* and *Ureaplasma*, are recognized bovine pathogens. Employing a diagnostic approach that targets the entire Mollicutes class, rather than focusing solely on the genus *Ureaplasma*, broadens the spectrum of potential pathogens, enabling the identification of co-infections or less expected agents with sanitary impact, thereby providing a more comprehensive basis for risk analysis.

Data were compiled in Microsoft Excel^®^ spreadsheets and checked for possible inconsistencies, resulting in 20 explanatory variables for the analysis. A univariate logistic regression model was used for the selection of variables, using a significance level of 20%, in the Generalized Estimating Equations (GEE), for the selection of variables that would compose the multiple model. The correlation structure defined in the model was exchangeable, in which the correlation of all observations is the same. Then, to select the multiple model, the backward selection method of the univariate analysis variables was used, and successively quasi-likelihood under the independence model criterion (QIC), considering a significance level of up to 5%.

Mollicutes detection was considered the dependent variable in the model, while the independent variables were herd size, average milk production, age group, breed, breeding system, quarantine, reproductive management, delivery yards, isolation and destination of animals with reproductive problems, fate of aborted fetuses, and history of presence of vaginal mucus, repetition of estrus, infertility, decrease in pregnancy rates, reproductive system lesions, cases of abort, stillbirth, weak offspring and placental retention. All analyses were performed using the R language [27].

## Results and discussion

The study revealed PCR positivity in 45.4% of cows for *Mollicutes* and 21.7% for *Ureaplasma diversum*, indicating the natural presence of these microorganisms in the genital mucosa of healthy animals. The prevalence per herd was 88.8% Mollicutes and *Ureaplasma diversum*. Table 1 presents results per herd. It is interesting to note that in practically all herds there was at least one positive animal. The herds that did not present positive animals had a low representation of individuals, which may have led to these results of undetected animals for the agents studied. The PCR detection rate was higher for the Mollicutes class, possibly due to the coexistence of other species, such as *Mycoplasma* spp. and *Acholeplasma* spp., in the genital mucosa of these animals [25]. On the other hand, Mollicutes can act as opportunistic agents with other bacteria or viruses, potentially leading to the onset of illnesses in stressful situations [7, 20].

**Table 1:** Results of prevalence per herd of mollicutes and *Ureaplasma diversum* using PCR in samples from reproductive tract of dairy cows

ID	Origin*	Mollicutes	Ureaplasma diversum
A	RJ	20.0% (2/10)	10.0% (1/10)
B	RJ	14.3% (2/14)	14.3% (2/14)
C	RJ	57.1% (16/28)	10.7% (3/28)
D	RJ	56.5% (13/23)	21.7% (5/23)
E	RJ	57.1% (8/14)	28.6% (4/14)
F	RJ	22.2% (4/18)	16.7% (3/18)
G	RJ	0% (0/2)	0% (0/2)
H	RJ	0% (0/6)	0% (0/6)
I	RJ	30.8% (8/26)	15.4% (4/26)
J	MG	53.6% (15/28)	35.7% (10/28)
K	MG	60.0% (15/25)	20.0% (5/25)
L	MG	100.0% (20/20)	25.0% (5/20)
M	MG	80.0% (16/20)	25.0% (5/20)
N	MG	12.0% (3/25)	24.0% (6/25)
O	MG	60.0% (18/30)	16.7% (5/30)
P	SP	35.0% (14/40)	20.0% (8/40)
Q	SP	33.3% (10/30)	26.7% (8/30)
R	SP	42.4% (14/33)	33.3% (11/33)

*RJ Rio de Janeiro state; *MG* Minas Gerais state; *SP* São Paulo state

The results of the univariate analysis are in Table 2, being selected variables for the multivariate method (*p* < 0.20). The multivariate model demonstrated a significant associating the detection of Mollicutes with various reproductive disorders variables in herds (*p* < 0.05). These variables include repeated estrus, infertility, placental retention and reproductive system lesions (Table 3). Besides, our analysis revealed that herds with over 100 animals, heifers and average milk production over 500 kg are also risk factors for Mollicutes detection (*p* < 0.05) (Table 3). Larger herds tend to concentrate a greater number of animals in close proximity, promoting the horizontal dissemination of the pathogen. Furthermore, exposure to physiological or pathological secretions and urine from carrier animals significantly increases the spread of Mollicutes within herds [17, 18]. Moreover, larger herds experience a higher volume of animal exchange through buying and selling, which increases the likelihood of failures in biosecurity practices on the property. This, in turn, raises the possibility of introducing and disseminating agents, often originating from asymptomatic individuals [13]. During the collection of samples, none of the animals exhibited signs of genital disease. However, it was observed that cows belonging to herds where vulvovaginal lesions were present had the highest risk of Mollicutes detection [30].

**Table 2:** Univariate modeling approach for risk analysis of mollicutes genital detection in dairy cattle herds from Southeast Brazil

Variable	Category	*N*	PCR Mollicutes (%)	OR	CI	*p*- value
Herd size	Until 100	145	35.2% (51)	1	1.074–6.545	0.034*
	> 100	247	51.4% (127)	2.652		
Average production	Until 500 kg	199	35.2% (70)	1	1.325–7.264	0.009*
	> 500 kg	193	56.0% (108)	3.103		
Age group	Heifers	91	70.3% (64)	4.612	1.692–12.568	0.003*
	Cows	301	37.9% (114)	1		
Breed	Holstein	108	60.2% (65)	3.375	1.039–10.963	0.043*
	Crossbreed	284	39.8% (113)	1		
Breeding system	Intensive	50	52.0% (26)	1.738	0.426–7.088 0.333–3.754	0.742
	Semi-intensive	312	43.6% (136)	1		
	Extensive	30	53.3% (16)	1.118		
Quarentine	No	265	47.2% (125)	1.190	0.532–2.660	0.672
	Yes	127	41.7% (53)	1		
Reproductive management	NM	141	37.6% (53)	1	1.007–13.489 0.457–2.745 1.266–4.956	0.002*
	AI + ET	98	62.2% (61)	3.685		
	NM + AI	130	39.2% (51)	1.120		
	ET	23	56.5% (13)	2.505		
Delivery yards	Present	284	38.0% (41)	1	0.667–4.667	0.253*
	Absent	108	48.2% (137)	1.764		
Isolation of animals with reproductive problems	No	244	45.9% (112)	1.041	0.451–2.405	0.925
	Yes	148	44.6% (66)	1		
Destination of animals with reproductive problems	Stay in herd	220	48.6% (107)	1.365	0.599–3.112	0.459
	Culling	172	41.3% (71)	1		
Fate of aborted fetuses	Discard	297	143 (48.1)	1.504	0.518–4.366	0.453
	Stay in the pasture	95	35 (36.8)	1		
Presence of vaginal mucus	No	190	37.4% (71)	1	0.874–4.658	0.100*
	Yes	202	53.0% (107)	2.018		
Repetition of estrus	No	145	33.1% (48)	1	1.182–4.597	0.015*
	Yes	247	52.6% (130)	2.331		
Infertility	No	177	33.9% (60)	1	1.111–4.893	0.025*
	Yes	215	54.9% (118)	2.331		
Decrease in pregnancy rates	Yes	63	33.3% (21)	1	0.574–6.111	0.285
	No	329	47.7% (157)	1.948		
Placental retention	No	131	28.2% (37)	1	1.437–7.405	0.005*
	Yes	261	54.0% (141)	3.262		
Reproductive system lesions	No	334	43.1% (144)	1	1.176–3290	0.010*
	Yes	58	58.6% (34)	1.967		
Abortion cases	No	139	38.1% (53)	1	0.714–3.564	0.255
	Yes	253	49.4% (125)	1.595		
Stillbirth cases	No	230	43.9% (101)	1	0.440–3.012	0.774
	Yes	162	47.5% (77)	1.152		
Weak offspring cases	No	308	40.6% (125)	1	0.641–8.034	0.204
	Yes	84	63.1% (53)	2.269		

* Significant associations (*p* < 0.20)

*OR* odds ratio; *CI* confidence interval; *NM* natural mount; *AI* artificial insemination; *ET* embryo transfer

**Table 3:** Final multi-model approach for assessing the risk of mollicutes genital detection in dairy cattle herds from Southeast Brazil

Effect	*p* value	OR	2.5%	97.5%
Intercept	0.000	0.152	0.080	0.290
Herd size over 100 animals	0.018*	2.083	1.134	3.823
Average production > 500Kg	0.025*	1.621	1.062	2.476
Age group Heifers	0.017*	4.006	1.283	12.510
Presence of vaginal mucus	0.053	0.424	0.177	1.011
Repetition of estrus	0.001*	2.854	1.513	5.418
Infertility	0.041*	0.471	0.229	0.970
Placental retention	0.000*	2.302	1.559	3.401
History of genital lesions	0.000*	5.564	3.011	10.284

* Significant associations (*p* < 0.05)

To determine the similarity level between the isolated *U. diversum* species in this study, we conducted Multiple alignments of the sequences targeting the 16–23 S rRNA internal transcribed spacer (ITS) region. The analysis revealed high similarities, with pairwise distances ranging from 96.32% to 100%, including the reference strains ATCC 49,782 (CP009770), T95 (JN935894), and the Brazilian *U. diversum* strain BRA/UEL3 (KJ767192) (Spreadsheet 1). These results confirmed that the isolates belonged to the *U. diversum* species and were deposited into GenBank under the accession numbers MT374127 to MT374154. Furthermore, a phylogenetic analysis was performed, which grouped the isolates into 16 clusters (Fig. 1). The cluster represented in blue was the most abundant, consisting of strains from São Paulo and Rio de Janeiro states. The second most populous cluster, represented in silver, included strains from all three surveyed states. Interestingly, the strains MSV_UFF01 and MSV_UFF09, grouped in the red cluster, originated from animals within the same institution that often relocates livestock animals for research purposes between these two states. The movement of animals between collection sites may explain the higher similarity observed within each cluster, as closely related strains are present in the vaginal microbiota of these animals. This finding suggests that the extensive animal movement has likely contributed to the spread of Mollicutes agents associated with reproductive disorders across the surveyed states.

To the best of our knowledge, this study represents one of the few investigations that have undertaken molecular characterization of *U. diversum* strains in Brazil using Sanger sequencing. The isolated strains in our study were derived from animals exhibiting no apparent genital lesions. Interestingly, we observed certain similarities between our strains and the ATCC 49782 strain, which has been associated with reproductive disorders in cattle such as placentitis, fetal alveolitis, abortion, and the birth of weak calves in Brazil [23]. This suggests a potential link between our strains and the aforementioned reproductive disorders. Moreover, it is worth noting that *U. diversum* may be present in both healthy and diseased cows, with the bacteria persisting longer in asymptomatic animals depending on the urea concentrations in the urogenital tract excreta, which serve as an essential nutrient for these bacteria. This silent disease can result in reduced reproductive performance in cattle and economic loss from *U. diversum* infection is reflected in bovine meat, milk, and semen production and marketing industries [21, 22, 31].

Few studies contemplate the molecular characterization of *U. diversum* strains. Vaginal swab samples from Brazilian cattle with GVV had the genetic sequences from the intergenic region evaluated [35], and showed a similarity with the sequences of the current study ranging from 96.32% to 100%, in which only animals without genital lesions were considered. Thus, it highlights that strains of *U. diversum* may be widespread and that they are also present both in healthy or sick cows.

## Conclusions

In conclusion, this study identified the presence of Mollicutes and *Ureaplasma diversum* in a significant proportion of dairy cows raised under tropical conditions, with associated risk factors identified for Mollicutes infection in various reproductive disorders variables, herds with over 100 animals, heifers and average production over 500 kg. The detection of different strains of *U. diversum* in multiple multiple locations suggests a widespread circulation of the agent likely facilitated by animal movement networks. The findings of this study highlight the importance of early detection and management of Mollicutes and *U. diversum* infections in dairy cattle to prevent economic losses and ensure animal welfare. Further research is needed to better understand the epidemiology and genetic diversity of these pathogens to inform targeted control measures.
